# The *Ethics of the Societal Entrenchment-approach* and the case of live uterus transplantation-IVF

**DOI:** 10.1007/s11019-019-09891-w

**Published:** 2019-05-17

**Authors:** Lisa Guntram, Kristin Zeiler

**Affiliations:** Department of Thematic Studies – Technology and Social Change, Linköping University, 581 83 Linköping, Sweden

**Keywords:** Uterus transplantation, Discourse, Societal entrenchment, Empirical ethics, Ethical problems, Surrogacy

## Abstract

In 2014, the first child in the world was born after live uterus transplantation and IVF (UTx-IVF). Before and after this event, ethical aspects of UTx-IVF have been discussed in the medical and bioethical debate as well as, with varying intensity, in Swedish media and political fora. This article examines what comes to be identified as important ethical problems and solutions in the media debate of UTx-IVF in Sweden, showing specifically how problems, target groups, goals, benefits, risks and stakes are delineated and positioned. It also demonstrates how specific assumptions, norms and values are expressed and used to underpin specific positions within this debate, and how certain subjects, desires and risks become shrouded or simply omitted from it. This approach—which we label the *Ethics of the Societal Entrenchment-approach*, inspired by Koch and Stemerding (1994)—allows us to discuss how the identification of something as the problem helps to shape what gets to be described as a solution, and how specific solutions provide frameworks within which problems can be stated and emphasised. We also offer a critical discussion of whether some of these articulations and formations should be seen as ethically troubling, and if so, why.

## Introduction

The first child in the world to be born after live uterus transplantation and IVF (hereafter referred to as UTx-IVF) was born in 2014, in Gothenburg, Sweden.^1^ Before and after this event, intense medical and bioethical debate has focused on risk/benefit analyses that concern possible implications for the uterus recipient, the intended child and, at times, the donor (see e.g. Arora and Blake 2014; Olausson et al. 2014; Testa and Johannesson 2017). Debate has also covered live *versus* deceased UTx donation; the equitable distribution of resources and access; reproductive liberty, and priority-setting (Alghrani 2018; Bruno and Arora 2018; Sandman 2018; Wilkinson and Williams 2016). Normative assumptions about such matters as what should be seen as acceptable and desired routes to parenthood and what should be considered acceptable risks of UTx-IVF have been discussed, as has a potential moral imperative of UTx-IVF (Allyse 2018; Catsanos et al. 2013; Guntram and Williams 2018; Lotz 2018).^2^

Ethical aspects of UTx-IVF have also been debated in Swedish media and political fora, with varying intensity. This article examines what comes to be identified as important ethical problems and solutions in the media debate of UTx-IVF in Sweden between 1998 and 2018. We identify three discourse coalitions within the material we have studied, i.e. three distinct ensembles of actors that draw on or embrace a certain discourse or sets of discourses, and that by so doing render specific problems and solutions as central. We show how problems, target groups, goals, benefits, risks and stakes are delineated and positioned. We also demonstrate how specific assumptions, norms and values are expressed and used to underpin particular positions within this debate, and how certain subjects, desires and risks become shrouded or simply omitted from the media discussions.

More specifically, this analytic approach allows us to discuss how the identification of something as *the problem* helps to shape what gets to be described as *a solution*, and how s*pecific solutions provide frameworks within which problems can be stated and emphasised* (Koch and Svendsen 2005). Further, we show that the development and introduction of UTx-IVF, as a new combination of technologies in Sweden, took shape in complex interactions of “societal entrenchment” (Koch and Stemerding 1994, p. 1212). Societal entrenchment, Lene Koch and Dirk Stemerding explain, refers to a process that includes further technological refinement, articulations of a demand or a need for the technology, and articulations of cultural and political acceptability (Koch and Stemerding 1994, p.1212). Such entrenchment can also include ardent critique.

The approach used in this article is based on Koch and Stemerding’s understanding of societal entrenchment and we label it the *Ethics of the Societal Entrenchment-approach*, since it specifically attends to ethical aspects. It addresses ethical aspects within and of societal entrenchment, analytically and critically, and it contributes to the field of empirical ethics.^3^ It engages critically with details of societal entrenchment in particular cases, with a focus on what ethical aspects of UTx-IVF are discussed, how these are discussed, and what gets to be talked about as central ethical concerns. Importantly, it does not just descriptively state the values and norms that are expressed within a particular context or situation, but analyses the underpinnings, articulations, discursive alliances, and formations—in short, the entrenchment—that allow certain ethical positions to gain authority. In a second step, we use our approach to offer a critical discussion of whether some of these articulations and formations *should* be seen as ethically troubling, and if so, why.

To examine the ethics within and of social entrenchment is to critically examine the conditions, processes, and relationships through which certain medical practices and ethical arguments take shape, and gain or fail to gain acceptance, in the concrete case when particular medical technologies are developed. We conclude that not to address these issues is to miss out on ethical pertinent features of the development of new medical practices, and of bioethics as articulated in public discussions. In order to spell out what we do differently from some other approaches to empirical ethics, we contrast our approach with the four levels of empirical ethics identified by Kon (2009): *Lay of the Land, Ideal versus Reality, Improving Care*, and *Changing Ethical Norms*.

## Social entrenchment, and the making of solutions and problems

The concept of “societal entrenchment” (Koch and Stemerding 1994, p. 1212) captures how the development and introduction of new technologies, or in our case a new combination of technologies, take shape in complex interactions between a variety of actors. In this process, technology is further refined; a demand or need for the technology is articulated; and cultural and political acceptability gradually gained (Koch and Stemerding 1994). However, this does not happen automatically. It requires activities and environments in which applications of the new technology can be realised, or, in other words, in which they can be “entrenched.”

Already existing networks and regimes—i.e. “mutually adapted and coordinated technological, organizational and societal practices” (Koch and Stemerding 1994, p. 1213)—that have taken shape through previous attunements of technological options, demand and acceptability, play a crucial role in the initial introduction of new technologies. Yet, the development and societal entrenchment of new technologies are not necessarily a smooth process, but is typically met with questions, concerns and resistance from various parties in light of their potentially adverse societal implications (Ibid.).

The concept of societal entrenchment can furthermore tie into examinations of how technological innovations create frameworks within which certain problems can be stated and handled and certain solution can be naturalized and be perceived as unproblematic (see also Koch and Svendsen 2005; Weiss 1987). Such examinations show how a novel solution and the understandings that underpin it can shape the way in which medical conditions are defined as problems and how they are to be solved (see Spector and Kitsuse 2001). Thus, solutions can be seen to shape the identification and formulation of appropriate problems (Whyte 2005).

In the first part of this article, we consider the understanding of societal entrenchment as outlined by Koch and Stemerding (1994) and the examination of problems and solutions as outlined by Koch and Svendsen (2005). This enables us to examine how ethical problems and solutions are expressed, and what are accepted as central ethical views and arguments. We see such empirically-oriented analytic work as the first step of the *Ethics ofthe Social Entrenchment-approach* that we propose in this article. In the second part, we engage critically with our results, and address what we see as ethically troubling features of the debate about UTx-IVF, based on our results from the first step. We also discuss why we think these should be seen as troubling, and why we consider that they need further ethical analysis.

## Situating UTx-IVF

### The Swedish context

Researchers at Sahlgrenska University Hospital, Sweden, have worked towards finding a way to transplant a uterus since the late 1990s, as a treatment for uterine factor infertility (UFI)^4^ (Brännström 2015). In 2012, the first woman in the Swedish UTx-IVF trial received a uterus from her mother (Brännström et al. 2014), and soon thereafter eight more women received uteri from live donors who were related to, or a friend of, the recipient.

Donors, recipients, and recipients’ partners were all extensively medically and psychologically screened before being allowed to take part in the trial. Eggs were collected from the recipient, and fertilized in vitro with sperm from her partner. When a satisfactory number of high-quality embryos had been stored, the cervix, the uterus and two major blood vessels were transplanted and connected to the vagina of the recipient. In the first nine transplantations, the duration of surgery ranged from 10 to 13 h for the donors and from 4 to 6 h for the recipients (Brännström et al. 2014). After surgery, the recipients were given immunosuppressive treatment to avoid rejection. It was, however, necessary to remove the uteri from two of the recipients due to rejection episodes soon after transplantation (Brännström 2018).

For those who started menstruating and whose uteri were considered stable, embryo transfer (ET) was initiated about a year after surgery. However, later on, it was reported that the number of ETs had ranged from 0 to 14 per couple (Kvarnström 2017). All births took place through caesarean section, and the first delivery was reported in 2014 (Brännström 2018). The protocol of the Swedish trial specified that if the recipient wished to and was assessed to be medically fit, a second pregnancy could be initiated. The protocol also specified that it was not intended that the transplanted uterus last for the lifetime of the recipient, but was to be removed after 5 years. This was done in order to reduce the time spent on immunosuppressant treatment (Gustavsson Kubista 2018). In 2018, it was reported that six women in the Swedish trial had given birth; four of them to one child each and two of them to two children each. Thus, the Swedish trial had resulted in eight children being born (Brännström 2018).

In Sweden, uterus transplantation has been and is performed and funded solely as a research procedure, and is not accessible through the general health care system. Neither donors nor recipients are compensated for loss of income, travel, accommodation, or other costs during hospital stay and sick leave. However, hospital care and examinations prior to and after transplantation are covered, and both donors and recipients are covered by the same insurance policy as all other patients treated in Swedish hospitals. They are, furthermore, entitled to “högkostnadsskydd” (Personal communication, Anna-Karin Thölin, 18 September 2018), which means that no patient needs to pay more than SEK 1150 (€ 115) per year for medical care (1177 Vårdguiden 2019).

If UTx-IVF were to be offered as part of general healthcare, the financial coverage might align with that applied in other forms of organ donation, and the economic compensation could be comparable to that of live kidney donation. Health care in Sweden is a public service funded through taxes, and the tax-funded healthcare and welfare system covers almost all costs related to end-stage renal disease treatment (see e.g., Wikström et al. 2007), even though there is a small co-payment. In 2017, a patient paid a maximum of SEK 1100 (€ 110) per year for medical care, a maximum of SEK 2200 (€ 220) per year for prescription drugs, and a maximum of SEK 1980 (€ 198) per year for travel to hospital. In live kidney donation, donors are financially compensated only for loss of income and other verified costs during hospital stay and sick leave.

Of significance for our analysis are also the Swedish policy and regulation of related practices such as altruistic surrogacy, IVF and adoption. While surrogacy is not specifically regulated, it is in practice forbidden since legislation specifies that only single women and couples who include a woman who can carry and give birth to an intended child can access fertility treatment (Ministry of Health and Social Affairs 2006).^5^ The same legislation results in IVF being accessible through the health care system for heterosexual couples, single women and lesbian couples (Ministry of Health and Social Affairs 2006). Although regional differences exist, most county councils cover three IVF cycles through the tax-funded healthcare system. Finally, married heterosexual couples, same-sex couples registered as partners, and single individuals are allowed to apply for adoption in Sweden. Inter-country adoption is the most common form of adoption. In 2013, 350 inter-country adoptions of children were carried out, which is to be compared with 45 national adoptions of children not related to the adoptive parent(s) (Socialstyrelsen 2014, p. 16).^6^

### Bioethical perspectives on organ donation and assisted reproduction technologies

Since UTx-IVF combines organ transplantation with IVF, the ethical debate on UTx-IVF resonates with concerns within these larger fields. Advances in transplantation medicine in general have often been portrayed as a most remarkable and miraculous medical invention (see e.g. Ambagtsheer et al. 2013; Gunnarsson 2016; Monaco 2007). Scholars have discussed the conditions for decision-making in relation to organ donation; the risk of undue pressure to donate; the meaning of different understandings and practices of consent in this area, and the pros and cons of different forms of consent (Crouch and Elliott 1999; Forsberg et al. 2004). Further, studies have shown how parents may feel that they should do what they can for their child, including donating, and how “excorporating” such assumptions can be experienced as emotionally turbulent (Zeiler 2018).

Other discussions have centred on the motivations of donors; on altruistic gift-giving versus market-like practices; on the role of money in these practices; and on how to increase the number of donors (for an overview of such discussions, see for example Malmqvist and Zeiler 2016). Further, scholars have emphasised that even if theoretical ethical matters have been considered, transplantation may have unintended and unexpected effects on donor-recipient relationships. For example, recipients can come to experience the new organ—from someone else—in their body as other than oneself and at times as troublingly so (see, as but some examples, Haddow 2005; Shildrick et al. 2009; Zeiler 2009). In the light of these analyses, the description of transplantation as miraculous has been questioned, and the risk that this description diverts attention away from the contingencies, complexities and suffering associated with the procedure has been pointed out (Crowley-Matoka et al. 2004; Gunnarson 2016; Kierans 2005; Sharp 2006; Waldby and Mitchell 2006).

Within literature that discusses the ethics of IVF, risk–benefit analyses are common. These typically emphasise that risks are low and that IVF has, after all, become an established practice in many Western countries (see, for example Marina et al. 2010). It is also commonly stated that IVF should take place only when the patients involved have been duly informed about the risks, have had the time and opportunity required to consider alternatives, and have made an informed decision. However, even if IVF has been described as generally acceptable to patients, some risks are involved, such as the risk of ovarian hyperstimulation syndrome. This is physically demanding and may be fatal (Delvigne and Rozenberg 2002). Research has also shown that IVF can evoke anxiety, because of, for example, its uncertain outcome (Verhaak et al. 2007). Scholars have addressed the complexity of the decision whether to undergo IVF, and the difficulty of imagining what it can entail before starting treatment (Franklin 1997; Kirkman and Rosenthal 1999; Throsby 2004). Other discussions have concerned whether IVF has predominantly emancipatory or more troubling effects for the women involved (Corea 1985; Sandelowski 1990; Rowland 1992; Ulrich and Weatherall 2000). Research has also shown that IVF can reinforce assumptions, values and norms about biological motherhood (Gentile 2013; Morell 2000; Rich 1995; Whiteford and Gonzalez 1995), and make possible new kinship constellations (Edwards et al. 1993; McKinnon 2015; Thompson 2001).

### Methodological considerations: materials and analysis

The data analyzed consisted of articles, opinion papers, and news pieces in printed Swedish newspaper media, in the period May 1998 to August 2018.^7^ The discourse analytic framework predominantly drew on Norman Fairclough’s work on critical policy studies (Fairclough and Fairclough 2013; Fairclough 2013), which discusses discourses and lines of argumentation, and on Hajer’s (1993) work on discourse coalitions.

A *discourse*, as we understand it, is a combination of “ideas, concepts, and categories through which meaning is given to a certain phenomenon” (Hajer 1993, p. 45). It “frame[s]” or shapes a problem in a specific way, and the ideas, concepts and categories that constitute it may also shape what becomes defined as the solution to the problem (Hajer 1993, p. 45). A *discourse coalition* refers to an ensemble of actors who draw on or embrace a certain discourse or certain sets of discourses. In a discourse coalition, interests, goals, and world views are not necessarily shared, but are based on common ways of understanding and assigning meaning to the issue at hand (Hajer 1996, 247). Differently put, the concept of a “discourse coalition” illustrates how actors who are not “singing in the same choir” nevertheless might “sing in chorus” (Szarka 2004, p. 319). Furthermore, an *argument* can draw on different discourses and take different forms. An argument may be presented by way of contrasts, for example, and it typically conceptualizes a certain aspect of the world in a particular way (Hajer 1993; compare Bacchi 2012).

We identified one dominant and two smaller, and more fragile, discourse coalitions. The first coalition consisted of the transplantation teams and their patients, who gave accounts to journalists. Within this discourse coalition, live UTx-IVF was described as *the* solution to the problem of UFI. The second discourse coalition was predominantly made up by medical professionals outside the transplantation team and medical ethicists, who wrote articles themselves, and women with UFI, who gave accounts to journalists. This coalition accepted the definition of the problem given by the first discourse coalition only partially: that women with UFI who wished to become genetic and gestational mothers had not previously been helped by the health care system, and this was problematic. Also the third discourse coalition was populated by medical ethicists, and by some columnists and journalists. This coalition re-defined the problem in terms of the limits of medical innovation and ethical concerns, such as the issue of priority-setting.

The analysis of discourse coalitions, discourses, and arguments made it possible to identify features of the societal entrenchment of UTx-IVF and its ethical aspects. The introduction and execution of UTx-IVF was discussed in different ways, was presented as solving different problems, and enabled certain problems to be stated and handled.

## UTx-IVF in Sweden: defining the problem(s), finding the solution(s)

### A solution in need of a problem: making UFI the problem that UTx-IVF solves. The first discourse coalition

In the summer of 2011, many Swedish newspapers reported about a historical surgical procedure that was about to be performed by a Swedish research team. For the first time, a mother was donating her uterus to her daughter (Asplind 2011; Åkerman 2011). This development was the result of several years of hard work in the lab and in animal models (Larsson 2003; Sundin 2003)—work that had made the researchers particularly highly skilled and now able to move on to UTx-IVF in humans, the reports stated (Hansson 2003; Kasvi 2012). Described as a “unique”, “ground-breaking” and “first in the world” event, UTx-IVF was framed as an unprecedented solution. Borrowing from Koch and Svendsen (2005), we ask; how did this framing of UTx-IVF as an *extraordinary* innovation contribute to shape the problem that UTx-IVF was meant to solve?

Throughout the first discourse coalition, the overarching message of the actors was clear: UTx-IVF was meant to deal with *one* specific problem, namely UFI. The description of the problem—that women with a non-functioning or with no uterus were unable to “have children” (Alvarsson 2012; Karlsson 2012; TT 2011) or to have a child of their “own” (Medicinsk Access 2014; Rogsten 2014; Svenberg 2014)—drew its force from the assumption that “having children is a central aspect to many women in the world” (Gisselquist 2014).^8^ This assumption thus squared well with contemporary discourses in which a desire for children is portrayed as an assumed dimension of a “normal” life course (Gentile 2013).

Accounts of the problem were also complemented with statistics on the prevalence of UFI. While it was occasionally noted that “no exact numbers on how many women are affected by uterine factor infertility are available” (William-Olsson 2002), or that UTx-IVF *might* become a solution for *some* of the women affected by UFI (Bratt 2012; Sims 2017), the statistics presented typically underscored how common the condition was. As some examples, actors in this coalition stressed that UFI affects as many as 1.5% of all women in the world (Hansson 2003); is shared by approximately 200,000 women in Europe (Tjernberg 2014); and affects thousands of women in Sweden alone (Gisselqvist 2014; Hillgren 2012; William-Olsson 2002). In some cases, the prevalence of UFI was equated with involuntary childlessness, so that references to statistics boosted the number of potential women who might want to alleviate their desire to have children and become pregnant through UTx-IVF. This framing of UFI as common problem contributed to articulating the demand for UTx-IVF.

In order to understand how the problem was defined in the first coalition, it is important to consider two more aspects. First, the problem was at times further specified as a *matter of biology* in accounts in which genetics and gestation were described as, or assumed to be, central to women with UFI. For example, it was stated that “alternatives for women without a uterus who want a biological child” have been lacking (adoption, then, was not considered to be an equal alternative) (Bratt 2012), and that UTx-IVF could enable women with UFI to “give birth” (Alvarsson 2012; Berglund 2009) or to “carry” a, or their “own”, child (Lagerwall 2001; William-Olsson 2002). Such accounts contributed to positioning UTx-IVF as not only *a* solution, but also *the* solution to UFI with respect to being able to provide something that alternatives such as surrogacy and adoption could not. As the desire to have “biological” children or children of “one’s” own was framed as common to women in general, such accounts also contributed to positioning UFI as equal to “any other” form of infertility. Second, accounts stressed that women with UFI was a group *suffering in silence* (Mattsson 2014; Westman 2015). Highlighting the impact that the problem had on the lives of those affected, such accounts contributed to articulating the demand, and to the acceptability of UTx-IVF, as they underscored how UTx-IVF contributes to relieving suffering. These three features—women’s desire for children, the role of biology, and suffering in silence—provided a framework for delineating UFI as the problem targeted by UTx-IVF.

We now turn to the solution to the problem of UFI. The first discourse coalition described UTx-IVF as the solution, and as equal to any other infertility treatment. This was clearly spelt out when the need of, and demand for, UTx-IVF was justified. This was the case, for example, when one person in this coalition underscored that if you happen to be born without a uterus, or happen to develop cancer at a young age, you “should be able to get treatments.” Others who were interviewed underlined that infertility, irrespective of the underlying medical condition, should be treated, and that IVF was quite common:

…childlessness is classified as a disease and we are supposed to cure disease. For example, at least a couple of percent of all children who are born today are testtube babies. (Berglund 2009, p. 22)

Furthermore, the “like any other” reasoning recurred in relation to the specific transplantation process. This was the case when UTx-IVF was compared to other forms of organ donation in ways that emphasised the ordinariness of the procedure. “[T]o us, there is nothing strange about me receiving my mother’s uterus”, one of the women who had been initially accepted into the trial at Sahlgrenska said, and continued, “[I]t’s like a kidney or any other organ” (Svanberg 2011). Similar analogies, but with respect to hysterectomies, were used when medical professionals described the risks of the procedure.

The surgery is no more risky than an ordinary hysterectomy, in which the uterus is removed. This is done 10,000 times every year. (Svanberg 2011)

By aligning UTx-IVF with ordinary hysterectomies and asserting the low risks of these,^9^ the risk of surgery in UTx-IVF was positioned to be similar to the risks of “any other” hysterectomy. The use of analogies thus helped to frame UTx-IVF as “simply” a matter of using *already well-known procedures and standardized techniques* in contemporary medicine—such as IVF, transplantation and hysterectomy. Enabling in this way the argument that UTx-IVF is medically quite uncomplicated, the use of analogies contributed not only to defend the procedure, but also to articulate its acceptability. Risks for donors—who would undergo surgery that at times lasted as long as 13 h—were not discussed.

However, the first discourse also drew on a “like no other” reasoning, which positioned UTx-IVF as a new and unique solution. Such accounts presented one of the medical professionals performing the transplantations, Mats Brännström, as “the miracle-maker” (Svenberg 2014). They also underlined that UTx-IVF was different from alternatives such as surrogacy and adoption. UTx-IVF was said to offer “hope” to a specific group of “childless individuals” (Aftonbladet 2007; Erfors 1998; TT 2001) for whom there previously “had been no treatment alternatives at all” (Pavlica and Rogsten 2014). Occasionally, UTx-IVF was specifically said to offer “opportunities to help these women to experience the joy of motherhood through transplantation” (Lagerwall 2001) and help women who have ovaries but lack a uterus to become real mothers (Funcke 2009; Tännsjö 2009). In this way, gestation was positioned as central to “real” motherhood. Further, UTx-IVF was envisaged as being “like no other” solution, both with respect to novelty and with respect to its unique deliverables (gestation and birth of a genetically related child).

Of particular relevance to our focus on ethical issues was the way in which the “like no other” reasoning was drawn on to claim that UTx-IVF could and did circumvent ethical concerns. This often took place in accounts in which UTx-IVF was differentiated from surrogacy. In such instances, the extraordinariness was emphasised, as UTx-IVF was positioned as *the more ethical* alternative. As an example, a doctor in the Swedish research trial was quoted as saying that women who act as surrogates often are “taken from” “exposed” countries. This was considered to be ethically troubling and helped to position UTx-IVF as the more ethical alternative (Kasvi 2012). Furthermore, and as a different ethical argument for UTx-IVF in preference to surrogacy, UTx-IVF was described as implying that the intended mother takes on all the risks associated with the IVF pregnancy, instead of a surrogate mother. Comparing in this way UTx-IVF with transnational surrogacy (and presumably to commercial surrogacy, though that was not explicitly stated), the first discourse coalition positioned UTx-IVF as ethically preferable to surrogacy.

Furthermore, while the “like any other” reasoning emphasised ordinariness, the “like no other” reasoning emphasised specificity. Together, they worked to justify the development of UTx-IVF, to claim its acceptability, and ultimately to present it as the preferred solution. This definition of the solution and the problem contributed to delineate a particular group of women with a specific need, which in turn—given the particular framework of desires, demand and biology—was presented as having a health care need that should be acknowledged and fulfilled by the healthcare system. In terms of ethics, the first discourse coalition focused on the benefits of UTx-IVF (relieving suffering, offering hope, meeting the desire for gestational and genetic motherhood), and on the relative benefits in comparison with surrogacy (in that UTx-IVF is assumed not to exploit women from “exposed” countries and not to shift the risks). UTx-IVF was thus presented not only as a novel but also as an unprecedented solution, both with respect to its deliverables and with respect to pressing ethical concerns.

### Challenging the solution and partly the definition of the problem. The second discourse coalition

The second discourse coalition only partially accepted the first coalition’s definition of the problem: that women with UFI who wished to become genetic and gestational mothers had not received help from the health care system to achieve this. The second coalition questioned in some respects the idea of genetics and gestation as central to motherhood, and questioned whether UTx-IVF was the solution to the problem as defined in the first coalition (Hallén et al. 2010).

Further, in contrast to the use by the first discourse coalition of “extraordinariness” to underscore the accomplishments of UTx-IVF, extraordinariness was used by the second discourse coalition to *emphasise the challenges, problems, and risks of UTx-IVF*. These were described as “extraordinary.” For example, the risks of the immunosuppressive treatment required were emphasized (Hallén et al. 2010; Hamberger 2012), and concerns were raised about the limited knowledge of pregnancy during immunosuppressive treatment, and about the fact that past knowledge had been acquired solely from pregnancy among kidney transplant patients (Hamberger 2012).

Concerns were also raised about UTx-IVF being “high-tech”, extremely complicated, and time-consuming. The procedure was described as involving substantial risks for both the child and the mother. The risk for uterus rupture was described as “not small”, and it was stressed that UTx-IVF would require delivery by caesarean section since vaginal birth would be too risky (Hamberger 2012). The probability that the child would be born prematurely, and the difficulty of assessing the risks (given that UTx-IVF was still conducted only within research trials) were further arguments that contributed to positioning UTx-IVF as extraordinarily problematic (Hallén et al. 2010; Johansson and Sahlin 2011). The second discourse coalition also pointed out, occasionally, that UTx-IVF might not be an alternative for all women with UFI. As an example, the first woman who gave birth as the result of UTx-IVF, was quoted saying that UTx-IVF “is not an easy choice for everyone”, and that you must be prepared for “a long process with many medical obstacles and hardships” and “have great hope, courage, inner strength and a determination to be able to go through that which is required of you” (Hansen 2015).

Furthermore, UTx-IVF was contrasted with surrogacy in lines of reasoning in which surrogacy was described as an alternative. On the one hand, some actors raised concerns about surrogacy being ethically more complex than UTx-IVF, given that it involved a surrogate mother who was expected to undergo the pregnancy and hand over the child after birth (Hallén et al. 2010). On the other hand, surrogate motherhood was described as already in use and established in other countries with good results (Hallén et al. 2010; Hamberger 2012). It was also emphasized that “thousands” of healthy children have been born after surrogacy in different parts of the world and that psychosocial studies show that these arrangements tend to function well (Hamberger 2012). Why, wondered, for example, one author, should not surrogacy and UTx-IVF *not* be considered on the same grounds? Specifically, she wrote,

We are genuinely happy when hearing that some women may have regained a uterus through an operation. Why can we not rejoice in the same way when it comes to the possibility for childless people getting help from a sister who wants to become a surrogate mother? (Wålsten 2012)^10^

Other actors within this coalition argued that surrogate motherhood was “considerably easier, safer, and cheaper” than live UTx-IVF (Hallén et al. 2010). Specifying the idea of safety, a medical doctor explained that surrogacy was “medically safer” (Hamberger 2012) and it was suggested elsewhere that there was no reason to financially support UTx-IVF when “the alternative is an easy political decision to allow surrogate motherhood” (Hallén et al. 2010, p. 107).

Further, the second discourse coalition also contrasted UTx-IVF with adoption. Two medical ethicists asked:

Does it have to be a child of one’s own, with one’s genes, conceived through IVF treatment, carried in a transplanted uterus and delivered by caesarean section? Is adoption ruled out? (Johansson and Sahlin 2011, p. 1348)

In spelling out the various steps of live UTx-IVF, but none of the various steps of adoption, this description could be read as positioning UTx-IVF as not such an easy route to the desired child, after all.

When different routes to a child were compared with each other, actors in the coalition were also concerned about the costs involved. Since it was expected that UTx-IVF would be costly, it was argued that it was necessary to discuss whether the procedure, just like adoption, should be paid for (at least partly) by those who want it (Lernfelt 2014). One ethicist was quoted saying:

I don’t question the desire for children, but it is important to weigh the risks of the research against societal benefits and maintain a critical stance to how money is spent. (Nasr 2012)^11^

In terms of ethics, the second discourse coalition focused on the risks for the mother and the child, and on some occasions described surrogate motherhood as a preferable route that involved less risk. UTx-IVF was said to be not worth the risks involved. Further, either it was assumed that the value of gestation does not outweigh the risks involved in UTx-IVF, or the value attributed to gestation was questioned. The use of contrasts enabled the value of gestation and/or genetics to be questioned. In these ways, this discourse coalition partly challenged the societal entrenchment—the making of UTx-IVF into something acceptable—articulated by the first discourse coalition.

### Challenging the solution and how it defines the problem. The third discourse coalition

The third discourse coalition levelled more radical critique: it challenged the way in which UTx-IVF was presented as a solution, and did not accept the description of the problem given by the first discourse coalition. While stressing the importance of acknowledging the pain that may be associated with involuntary childlessness, the third coalition emphasized that the problem concerned more general questions regarding potentially eroding values and priority-setting within the context of Swedish health care.

As an example, it was suggested that “we” might want to might want to consider the drivers in research and how we allocate the skills and expertise of pioneering researchers. “I understand…” wrote a columnist

…that such ground-breaking research [as UTx-IVF] is exciting for the doctors who travel all over the world to talk about their results. But, we still need to be able to discuss whether some of the best doctors that we’ve got should devote themselves to such research when there are so many sick who need help. (Norrman 2015)

In a stronger way than merely contrasting UTx-IVF with surrogacy or adoption, the third coalition questioned the very focus on infertility in the other definitions of the problem given by the two other coalitions.^12^ The problem in the third discourse coalition instead became a matter of how to handle a potential technological and moral imperative, i.e. whether all that can be done should be done, and why UTx-IVF was allowed in the first place (albeit within the limited scope of a research study). As an example, actors in this discourse coalition used metaphorical language in such questioning, one example of which is the description by medical ethicists of UTx-IVF as a case that seemed to illustrate a “moral compass that swings chaotically” in the “flux of a strong magnetic field” of desire to establish a new innovative medical technology (Johansson and Sahlin 2011). As other examples, the ethical debate about UTx-IVF was problematized through the contrasting effect of historical examples. This was the case when references were made to “dark chapters” in the history of science and medicine—such as the experiments conducted in the concentration camps during the Second World War, the Tuskegee study, and the Swedish “Vipeholm” caries experiment in which patients (among them children and adults with cognitive conditions) were given confectionery in a study conducted between 1945 and 1955, without consent being sought or given (see Bommenel 2006). There is a risk, one ethicist was quoted saying, that

…if we look back to too great an extent, and only point to the atrocities of the Nazis, we miss out on the challenges that we face ourselves. (Gunther 2015, p. 19)

As one example, an ethicists was also quoted saying, in relation to the UTx-IVF trial in Gothenburg:

In that case you have exposed a vulnerable group – women who very much want to have children – to a particularly experimental treatment. (Gunther 2015, p. 19)

In this manner, the third coalition underscored that something was off-track when it comes to the ethical considerations in the development of UTx-IVF (see also Borelius 2014). It may seem to be an ordinary medical development, but when investigated more carefully, the third coalition underlined, UTx-IVF is a clear example of the problem that a high-tech, high-profile medical innovation may dazzle us. Two further points were particularly noteworthy in this discourse coalition. First, concerns were raised about the power of narrative, and the way in which it determines whose needs are acknowledged. This was the case when actors contrasted the attention given to women with UFI, and the success stories of UTx-IVF, with the lack of attention to more mundane medical conditions. Actors in the third discourse coalition asked, for example, where the voices of people with dementia, another group that suffers, were in the public debate (Johansson and Sahlin 2011). That narratives of people with dementia rarely end up in the media spotlight, the same authors emphasised, does not mean that they are not suffering and may not have great or very great needs. Those voices should also be heard, but only some narratives, given by some people, make it into the public media (see also Hægerstam 2013). In contrast to the first coalition, which raised the concern that women with UFI are a group who has “suffered in silence”, the third coalition positioned the narratives of UFI as loud, and as being given space (or taking space). By raising concerns in this way that the power of some narratives is stronger than that of others, the third coalition suggested that infertility narratives drown out those of other vulnerable groups. The wish for UTx-IVF was thus—once again—positioned as extraordinary, but in this case in the sense that it may crowd out other health care needs.

Second, the third discourse coalition challenged previous and future investments in UTx-IVF. It engaged with the questions of how to set priorities, what should be included in the state-funded health care system, and why. One example is an article that mentioned that it had not been clarified what an UTx-IVF treatment (including IVF treatment, monitoring of pregnancy and caesarean section) costs, nor the costs of the extensive research project. The “first surgeries are *allegedly* [our emphasis] covered within the budget of the research project” (Haldesten 2012, p. 2), it was pointed out, which indicated uncertainty as to whether the costs of UTx-IVF development affect public health care after all.

In other instances it was indicated that UTx-IVF could not be completely disconnected from tax revenues and public health care, and should be seen as intrinsically associated with societal costs. In this way, one columnist (Norrman 2015, p. 17) noted that:

…staff and health care have been financed by a private funding body. But it will, of course, still generate costs for society.

These accounts of the costs associated with the development of UTx-IVF illustrated an uncertainty as to whether UTx-IVF competes with other treatments. Its funding scheme was here used to position the Swedish UTx-IVF development as an extraordinary case, that should be carefully considered when engaging with priority setting. Similarly, comparisons were drawn between what a certain amount of money could achieve in the clinic, when not used for UTx-IVF. Examples given included treatment for children with cancer or heart disease, and elderly people with dementia. When making financial assessments, it was argued, we must take into account not only the costs for a single transplantation, but also the total costs for developing the new technology (Johansson and Sahlin 2011).

The examples given above show how the third coalition urged that UTx-IVF be judged “like any other” treatment, and that we should take care that we are not lured by its spectacular framing and powerful narratives. The examples also tie into the more general issue of how priorities are to be set, and the core question raised by the third coalition: Should all that can be done be done? (Haldesten 2012). “I might be mocking and provocative”, wrote the medical editor-in-chief at Läkartidningen, and continued:

but I do believe it is valid to raise the question of whether all that can be done also should be done. It is the task of funders and of society to determine where the limited resources in research and health care are to be placed. There are many urgent areas, and not all of them are as spectacular and glamourous? (Östergren 2011, p. 4)

Likewise, concerns were raised whether all medical innovations should be covered by the general health care insurance. If we are to fund health care in solidarity, one columnist argued, it “…must be directed by need, not by demand, and everything that is possible may not be proper or reasonable. Especially not at the tax-payers’ expense” (Lann 2012, p. 2). While acknowledging that UTx-IVF is an interesting scientific advancement, this line of argument asserted that this does not necessarily imply that it should be introduced as a part of general healthcare. It was also argued that such an introduction would require an ethical analysis of the risks, benefits, needs and costs (Lynøe 2016).

The third discourse coalition challenged the selfevident acceptability of UTx-IVF as part of the Swedish health care system, explicitly and implicitly. In terms of ethics, this discourse coalition prescribed caution before investing in novel therapies such as UTx-IVF, and questioned the idea of a technological imperative. Calls for caution were also evoked about the risk of attending to that which can be perceived as new and exciting—rather than the more mundane everyday needs that cannot be as easily framed in this way. This coalition was concerned with whose voices were being listened to, and called for reflection on the possible drivers and motives behind certain medical research and priorities. Finally, concerns were also raised about the limits of medical innovation and how to set priorities in state-funded health care.

### Critical reflection in the *Ethics of the Societal Entrenchment-approach*: what we see as ethical concerns, and why

We have above identified what gets to be the problems and solutions in the three discourse coalitions, in the Swedish debate over UTx-IVF. The introduction and practice of UTx-IVF as presented, discussed and argued for in the Swedish media are part of complex interactions in which actors present different problems, solutions, demands and needs for the technology. Some actors also present critical concerns and arguments against the acceptability of UTx-IVF at the experimental level, and as part of Swedish health care in the future. The societal entrenchment, in other words, is not smooth. Resistance and questions have been voiced.

The first step of the *Ethics of the Societal Entrenchment-approach* was to analyse how this has taken place, and the problems, solutions, alliances, desires, concerns and arguments involved. We now turn to the second step of our proposed approach, and address five aspects of these debates that we find to be troubling in the light of the results of our analysis.

First, we see the comparisons between UTx-IVF and surrogacy as problematic. The referrals made to surrogacy in the material analysed are often vague. It is not specified what kind of surrogacy arrangement is intended: commercial surrogacy or non-financially rewarding intrafamilial surrogacy. However, if UTx-IVF and surrogacy are to be compared, the relevant comparison—in the light of how UTx-IVF is performed in Sweden—seems to be between non-financially rewarding intrafamilial and friend-to-friend UTx-IVF and non-financially rewarding intrafamilial and friend-to-friend surrogacy arrangements. However, statements such as surrogates often being “taken from” “vulnerable” countries indicate that the surrogacy arrangement involved in the comparison with UTx-IVF, in such reasoning, is a commercial arrangement. If that is the case, it can, of course, still be argued that UTx-IVF is ethically preferable to commercial surrogacy. However, very few voices, if any, have argued that commercial surrogacy be allowed in Sweden. Comparing UTx-IVF with a form of surrogacy that commonly is critizised, and not with intrafamilial non-commercial surrogacy that some have argued for (SMER 2013), might enable certain kinds of conclusions - while leaving out some other ethically relevant comparisons.

Second, we find comparisons between hysterectomies—considered to be very common and unproblematic—and uterus donation to be troubling, as such comparisons fail to account for the knowledge gaps with respect to women’s experiences of hysterectomy in general (Gelder et al. 2005; Solbrække and Bondevik 2015; Williams and Clark 2000). Further, and even more importantly, the way in which analogies with hysterectomies framed UTx-IVF as “simply” a matter of using *already well-known procedures and standardized techniques* in contemporary medicine diverts attention away from the contingencies and complexities associated with the experience of not only removing but also donating one’s uterus to someone close to you. Such framings, we argue, are troubling as they tend to gloss over the donors’ lived experiences of donation, and the risks, concerns and difficulties associated with donating this body part. In a similar manner, we find the way in which statistics about the number of women affected by UFI are used to emphasise the need for UTx-IVF to be troubling. Equating the absence of a uterus with being involuntarily childless risks glossing over the fact that not all women with UFI desire UTx-IVF. This equation, we hold, is problematic, since it reinforces ideas about an unmet need for UTx-IVF and assumptions about women’s reproductive desires and the desire for a uterus although research into the perspectives of women with UFI is very scarce (see however Guntram 2018). 

These first two aspects relate to a rhetorical staging that can render nuanced discussions difficult. Of course, the media might enact specific media dramaturgies, but for the ethical debate, we see this as unfortunate. Ethically relevant nuances risk being lost.

Third, in the light of Swedish health care being based on the idea of equal provision and access to health care (Government Offices of Sweden 2016), we find it troubling that certain subjects and concerns are not, or only occasionally, taken into consideration in the Swedish debate over UTx-IVF. Our analysis shows that male-to-female trans persons, who might desire a uterus as part of sex-confirming surgery (Alghrani 2018; Spillman and Sade 2018), are mentioned only occasionally (see Funcke 2009; Ny 2017; Tännsjö 2009). While there may be specific reasons to exclude UTx-IVF for trans persons from state-funded health care, the reasons for such exclusion must be discussed when a combination of technologies such as those involved in UTx-IVF is being developed.

Fourth, our analysis shows that the perspectives of donors and partners of women with UFI were rarely considered. The lack of such discussions makes clear the assumptions that the actors have made about who is exposed to the risks and benefits of UTx-IVF. While risks to those receiving the uteri through transplantation of course are central, the lack of discussion of possible implications on behalf of other persons—such as donors—can reinforce ideas about who is to be considered in the ethical debate over UTx-IVF. The exclusion of the voices of both some persons who might want to use UTx-IVF and those who might, implicitly, be assumed to not play as central a role in the latter parts of the execution of UTx-IVF (such as donors) can be understood in terms of power and inclusion: whose narrative gets to be heard in the public space? If the media wants to address ethics in a nuanced and careful way, such dimensions must be addressed. The absence of these voices in the debate is ethically troubling.

Fifth, and tying into the previous points, we find the recurring persistent lack of nuance with respect to various perspectives that engage with lived experiences of organ donation and assisted reproductive technologies to be ethically troubling. To fail to consider such experiences, from different perspectives, may centre the debate onto the claims for or against UTx-IVF, and there is a risk that ethical issues that only become apparent when engaging with such lived experiences and meaning-making are lost. For example, even though kidney donation is a standardized procedure, it can still be experienced in different ways, and not simply as “easy” (see, for example, Gunnarson 2016).

## *What the Ethics of the Societal Entrenchment-approach* helps to do

Finally, how does the approach proposed by this article contribute to empirical ethics? As an interdisciplinary field of inquiry characterized by its eye for values, norms, and ethical dimensions of medicine and the life sciences, bioethics has for long been fuelled by debates about whether to reconcile more descriptively oriented and more normative approaches, and if so, how (see, for example, Borry et al. 2005; Haimes 2002; Herrera 2008; Ives and Draper 2009; Zeiler 2005).^13^ Within the bioethical subfields of empirical philosophy and empirical ethics, several ways of combining empirically oriented research with philosophy or ethics exist.

Kon’s differentiation of four levels of empirical ethics (Kon 2009) is one way to describe this burgeoning field. We see his differentiation as a useful starting point for a discussion of what the *Ethics of the Societal Entrenchment-approach* helps to do differently. We also see his differentiation as useful, since it exemplifies a common understanding of empirical work as descriptive (as in *Lay of the Land* and in *Ideal versus Reality*), and exemplifies normative analysis as critically engaging with the results of the descriptive work. In contrast to this understanding, we share the view of, among others, Erica Haimes, who argues that this is an over-simplistic division between descriptive and normative ethics. Careful empirical work, Haimes (2002, p. 91) argues, can “contribute not only to the understanding of ethical issues but also to the understanding of the social processes through which those issues become constituted as ethical concerns.” Arguably, the question of what ethical issues get to be understood as central (through certain social processes) should matter to bioethics - as this can help delimit the field of inquiry in the first place.

Following Haimes’ suggestion, our approach brings together the more descriptive and critical dimensions, and this allows us to tease out other, often contextual, dimensions of ethical problems and challenges. Within the first step of the *Ethics of the Societal Entrenchment-approach*, we showed how discourse alliances were formed, and how these alliances positioned certain concerns as central ethical concerns. This is not just description; this is analysis. In the second step, we took this reasoning one step further and (albeit briefly) discussed aspects that we see as ethically troubling in the media discussions of UTx-IVF in Sweden.

In order to explain further what we see as the analytic value of the *Ethics of the Societal Entrenchment-approach*, we conclude the article by contrasting this approach with the four levels outlined by Kon (2009). Kon distinguishes between *Lay of the Land* studies that aim to describe or explain current practices, attitudes, opinions or preferences and in this way offer input that can help to improve healthcare practice or patients’ decision-making, and *Ideal versus Reality* studies that examine possible gaps between ethical norms, values or policies, on the one hand, and what takes place in practice, on the other hand.

Our *Ethics of the Societal Entrenchment-approach* offers an analysis of the media debate. In contrast to Kon’s distinction of *Lay of the Land* studies, our approach examines and problematizes how the development and introduction of a new combination of technologies take shape in complex interactions between a variety of actors. This analysis demonstrates how values, norms, and discourses are established and questioned in this process, and how certain actors or voices establish, or seek to establish, themselves as proponents for, or critics of, specific understandings of certain medical practices as ethical. We also show how certain ethical questions come to be positioned as important and other as not being important, and by whom.

Furthermore, the *Ethics of the Societal Entrenchment-approach* does not primarily target ideal versus reality: its aim is not to examine gaps between ideal and actual practices. Instead, it shows how different problems and solutions come to gain acceptance or be questioned, and what are considered to be ethically relevant aspects to address within a particular debate. It also critically discusses whether some questions or some ways of addressing certain questions should be positioned as ethically troubling and if so, why. In this way, it is different from *Ideal versus Reality*-studies.

Kon (2009) also distinguishes between *Improving Care* studies which aim to find ways to solve or minimize problems identified in *Ideal versus Reality* studies, and *Changing Ethical Norms* studies, which tend to build on all past levels of studies to recommend that certain ethical norms be changed. Our focus is not primarily on improving care, and while we are concerned with some aspects of the UTx-IVF debate in Sweden, our *Ethics of the Societal Entrenchment-approach* also differs from the *Changing Ethical Norms* approach of Kon. As shown above, we analyse the stakes in the debate, how these take shape and are justified, and who gets to have a say in the understanding of UTx-IVF as ethically acceptable or not. This allows us to address not only who enters into discourse coalition with whom, but also who is excluded. The approach allows us also to discuss whether this should be perceived as ethically troubling and if so, why.

In contrast to the *Changing Ethical Norms* approach, which draws on a large set of empirical studies that range across different practices, we stay close to the concrete case. In doing so, we demonstrate and critically discuss how the problem–solution logic is, at times, reversed, and what this means. At stake is not simply first a problem and then a solution, but something much more complex. If certain solutions, which help to formulate certain problems in need of specific solution, are developed first, bioethics must critically examine this very process, and this requires an analysis that goes beyond Kon’s four levels.

Finally, societal entrenchment studies, including the *Ethics of the Socieal Entrenchment-approach* that we have proposed, examine the co-emergence of science, medicine, and socio-cultural acceptance and questioning, with a focus on how a certain medical practice comes to be developed and, eventually, perhaps also accepted. Yet much bioethical analysis still takes place when a medical practice is already in place. However, once a certain practice has been accepted, in the sense of having become integrated into health care practice, it becomes much more difficult to close it down. We therefore contend that to *not* examine societal entrenchment is ethically problematic. To engage, critically and analytically, with the entrenchment through which certain questions and understandings become positioned as central ethical questions or understandings is crucial in the striving for ethical analysis of the development and introduction of new technologies.

## Footnotes

1 Previously, two uterus transplantation attempts had been carried out. In the first of these, performed in Saudi Arabia in the year 2000, a woman received a uterus from a non-related live donor. Three months later it was necessary to remove the organ because of graft failure (Fageeh et al. 2002). The second attempt, in Turkey, involved a deceased donor and has up until now resulted in two pregnancies, both, however, with early miscarriages (Erman Akar et al. 2013).

2 Scholars have also suggested that (bio-)intimacy “plays a vital part of the ethics of uterine exchanges” as they become ethically legitimized by “being embedded in already organized structures of intimacy” (Kroløkke and Petersen 2017, p. 207).

3 Empirical ethics is a large field. For descriptions and justification of such work, see, for example, Borry et al. (2005), Haimes (2002) and Zeiler (2005).

4 In the medical scientific literature (Brännström et al. 2015), uterine factor infertility is defined as infertility ‘caused by absence or dysfunction of the uterus’ due to ‘congenital or acquired reasons’ resulting from (i) earlier hysterectomy, (ii) congenital deformities, or (iii) adhesions in the uterine cavity, all which result in these women having no or a non-functioning uterus.

5 Women with uterine factor infertility cannot carry a pregnancy, but they may have genetic children through surrogate arrangements if they have functioning ovaries.

6 The average cost for an inter-country adoption is SEK 250,000, of which SEK 75,000 may be reimbursed by the Swedish Social Insurance Agency (SVT 2016).

7 The material was collected through the search engine Retriever Research. The first item on UTx-IVF was published in 1998, and we have therefore focused our search on the period of May 1, 1998–August 1, 2018. The search covered all printed pieces including the phrases “uterus AND transplantation” and/or “uterus transplant*” (in Swedish: “livmoder AND transplantation” and/or “livmodertranspl*”) and resulted in 376 items. While the majority of these simply reported about the development and on the stances taken by various actors, some expressed a specific stance and positioned this stance relative to those of other actors. All of the analyzed material had been published in Swedish and all translations in this article were made by L. Guntram.

8 The scope was occasionally widened to include individuals with trans experiences, see e.g. Funcke (2009), Ny (2017) and Tännsjö (2009).

9 Similarly, the risks associated with immunosuppression drugs during pregnancy, a concern often raised against UTx, was accounted for with reference to research on pregnant women with transplanted kidneys (William-Olsson 2002).

10 See also Jernbeck et al. (2011).

11 Related to this, another ethicist was elsewhere quoted as describing difficulties in assessing the costs (Alvarsson 2012, p. 17).

12 Some articles stand out from this focus. In a discussion piece in Dagens Nyheter a professor in philosophy includes uterus transplantation as an example of surgery in individuals with trans experiences in a discussion of whether individuals can choose their own sex and to what extent such a choice, and the extension of our possibilities to choose in general, is desirable (Tännsjö 2009). Defined as such, the problem differs somewhat from the focus on priority-setting and the limits of medical innovation found in accounts from others who are members of the third coalition. See also Funcke (2009) and Ny (2017).

13 As an example, scholars have argued that an understanding of the context and processes in which ethical questions arise is important (Haimes 2002; Hoeyer and Tutton 2005).
